# The influence of preferred place of birth on the course of pregnancy and labor among healthy nulliparous women: a prospective cohort study

**DOI:** 10.1186/s12884-015-0455-x

**Published:** 2015-02-14

**Authors:** Tamar M van Haaren-ten Haken, Marijke Hendrix, Luc J Smits, Marianne J Nieuwenhuijze, Johan L Severens, Raymond G de Vries, Jan G Nijhuis

**Affiliations:** Research Centre for Midwifery Science Maastricht, Zuyd University, PO Box 1256, 6201 BG Maastricht, The Netherlands; Department of Epidemiology, CAPHRI School for Public Health and Primary Care, Maastricht University Medical Centre, PO Box 616, 6200 MD Maastricht, The Netherlands; Erasmus University Rotterdam, Institute of Health Policy and Management, PO Box 1738, 3000 DR Rotterdam, The Netherlands; Maastricht University Medical Centre, CAPRHI School for Public Health and Primary Care, PO Box 616, 6200 MD Maastricht, The Netherlands; Department of Obstetrics & Gynecology/GROW – School for Oncology and Developmental Biology, Maastricht University Medical Centre, PO Box 5800, 6202 AZ Maastricht, The Netherlands

**Keywords:** Models of maternity care, Place of birth, Course of pregnancy, Birth outcomes

## Abstract

**Background:**

Most studies on birth settings investigate the association between planned place of birth at the start of labor and birth outcomes and intervention rates. To optimize maternity care it also is important to pay attention to the entire process of pregnancy and childbirth. This study explores the association between the initial preferred place of birth and model of care, and the course of pregnancy and labor in low-risk nulliparous women in the Netherlands.

**Methods:**

As part of a Dutch prospective cohort study (2007–2011), we compared medical indications during pregnancy and birth outcomes of 576 women who initially preferred a home birth (n = 226), a midwife-led hospital birth (n = 168) or an obstetrician-led hospital birth (n = 182). Data were obtained by a questionnaire before 20 weeks of gestation and by medical records. Analyses were performed according to the initial preferred place of birth.

**Results:**

Low-risk nulliparous women who preferred a home birth with midwife-led care were less likely to be diagnosed with a medical indication during pregnancy compared to women who preferred a birth with obstetrician-led care (OR 0.41 95% CI 0.25-0.66). Preferring a birth with midwife-led care – both at home and in hospital - was associated with lower odds of induced labor (OR 0.51 95% CI 0.28-0.95 respectively OR 0.42 95% CI 0.21-0.85) and epidural analgesia (OR 0.32 95% CI 0.18-0.56 respectively OR 0.34 95% CI 0.19-0.62) compared to preferring a birth with obstetrician-led care. In addition, women who preferred a home birth were less likely to experience augmentation of labor (OR 0.54 95% CI 0.32-0.93) and narcotic analgesia (OR 0.41 95% CI 0.21-0.79) compared to women who preferred a birth with obstetrician-led care. We observed no significant association between preferred place of birth and mode of birth.

**Conclusions:**

Nulliparous women who initially preferred a home birth were less likely to be diagnosed with a medical indication during pregnancy. Women who initially preferred a birth with midwife-led care – both at home and in hospital – experienced lower rates of interventions during labor. Although some differences can be attributed to the model of care, we suggest that characteristics and attitudes of women themselves also play an important role.

## Background

Studies of place of birth have consistently shown lower rates of intervention in labor and birth for women with low-risk pregnancies who planned their birth at home [1-7]. Similarly, research confirms that when compared to other models of maternity care, midwife-led care reduces the rates of intervention in labor [1,4,5,8,9]. While these studies are convincing, maternity care is complex, and it is difficult to consider the degree to which the likelihood of intervention is influenced by birth setting, the philosophy of the care provider, or the characteristics and attitudes of the women. Klein et al. [10] showed in their study that women using midwife care consistently reported attitudes supporting less frequent use of technology compared to women receiving care from obstetricians. It also is probable that midwives will be less likely to intervene due to their philosophical and physiological orientation toward childbirth [11]. On the other hand, some studies comparing home and hospital birth with the same midwives providing care in both settings found lower intervention rates in the home birth group, suggesting that the birth setting also has a significant effect on outcomes [2,5]. The outcome measures used in most studies of birthplace and models of maternity care are obstetric intervention rates and birth outcomes [1-6,8,9]. In addition, most of these studies used planned place of birth at the onset of labor [1-6,8]. However, most women express a preference for a specific birth setting (model of care and place of birth) during pregnancy, long before labor begins [12]. Little is known about the influence of these early preferences on the course of pregnancy, labor, and childbirth.

The aim of our study was to explore whether the initial preferred place of birth at the onset of pregnancy *– i.e. home or hospital -* and model of care *– i.e. midwife-led care or obstetrician-led care –* are associated with differences in the course of pregnancy, intrapartum interventions, and birth outcomes in low risk nulliparous women in the Netherlands. By using the initial preferred place of birth instead of the actual place of birth we are able to gain insight into the influence of women themselves – i.e., their characteristics and attitudes – on the course of childbirth.

The course of the prenatal period is influential in determining the final birth setting and the management of labor. If policy makers and health care providers want to optimize maternity care, they must consider not only the outcomes of birth, but also the entire process of pregnancy and childbirth. Because there is a well-integrated, nationwide maternity care system, where home and hospital birth are both seen as a normal setting for giving birth, the Dutch environment is an ideal setting for studies on place of birth. Dutch maternity care is based on the principle that pregnancy and childbirth are fundamentally physiologic processes [13]. Independent practicing midwives provide care to healthy women with uncomplicated pregnancies, referred to as ‘midwife-led care’. Midwives refer women to obstetrician-led care when there is an increased risk of complications, as defined by the ‘List of Obstetric Indications’, a national guideline developed cooperatively by all the professions involved in maternity care [13]. We refer to this as a medical indication for obstetrician-led care. When in obstetrician-led care, a woman may receive care from a clinical midwife or an obstetric resident, but the supervising obstetrician has the overall responsibility for the care. Women with a low-risk pregnancy are free to follow their preferences and give birth at home or in hospital under the supervision of the independent midwife. In midwife-led care, women will not receive medical interventions such as medical pain relief, augmentation, or continuous fetal monitoring. Women will receive these interventions if necessary, but only after a referral to obstetrician-led care. If a healthy woman prefers a midwife-led hospital birth, she is charged a co-payment of approximately € 300 (US$ 410) for the additional cost of the hospital stay, a charge that some, but not all, insurance plans cover. Although uncommon, access to obstetrician-led care is possible when low-risk women have a strong preference for giving birth under the supervision of an obstetrician. At present, no co-payment is required for an obstetrician-led low-risk birth in a hospital. It is likely that this is a result of the fact that it is quite unusual for a low-risk woman to have an obstetrician supervise her birth, and thus insurance companies assume these women must have some medical indication for obstetrician-led care. The exact number of low-risk women whose primary choice is obstetrician-led care is unknown.

## Methods

### Study sample

We conducted a multicenter, prospective cohort study among low-risk nulliparous women who started their pregnancy in midwife-led care or in obstetrician-led care. Of the 466 independent midwifery practices in the Dutch Midwifery Association Registration in 2006, we randomly selected and invited 150 practices from across the Netherlands to recruit women in midwife-led care. One hundred practices, including rural and urban areas, agreed to participate. The reason most often given for not participating in the study was a lack of time. There is no evidence that the non-participating practices differ significantly from those willing to participate. Of the 90 hospitals with maternity care units in the Netherlands, 30 hospitals were randomly chosen and asked to recruit low-risk women in obstetrician-led care. Fourteen hospitals, three academic and 11 non-academic, agreed to participate. Most frequently given reasons for non-participation were too many other on-going studies and the expectation of too few suitable participants for this study, as midwife-led care is the norm for low-risk women in the Netherlands. Participating midwifery practices and hospitals received 25 information packs including project information and an informed consent form and were asked to distribute these to pregnant women who met the inclusion criteria during the first consultation at 8–12 weeks pregnancy. Information on the project contained the background and purpose of the study, the procedures involved in the study, the possible risks and benefits of taking part in the study and the rights of the participants. Eligible women who received information from their caregiver were asked whether the researchers could contact them by telephone to give further information about the study. Women who agreed were called by the researchers, received more information if required, and were formally asked to participate. A signed informed consent form was required for all participants. Women with a first on-going pregnancy and without an obstetric or medical indication according to the List of Obstetric Indications were included. We enrolled only women expecting their first birth so that their previous birth experiences would not affect their preferences and outcomes. Recruitment in midwifery practices was carried out from March 2007 to August 2007 and in hospitals from March 2007 to December 2011. The longer inclusion period in the hospitals was necessary because, as noted above, it is not a common practice for low-risk women to have obstetrician-led care. All women gave informed written consent to participate, and ethical approval was obtained by the Medical Ethical Committee of the Maastricht University Medical Centre (registration no. 04–234/11-4-009).

### Data collection

Our data were collected using self-reported questionnaires, medical records and birth registration forms. Sufficient knowledge of the Dutch language was required to read and fill out the questionnaires. All women completed a questionnaire – by post or online - before week 20 of their pregnancy. The questionnaire was pre-tested in three midwifery practices. Women were asked to indicate which place of birth they preferred: a midwife-led home birth, a midwife-led hospital birth, an obstetrician-led hospital birth, or ‘I do not know yet’. Additionally, the questionnaire included questions about socio-demographic and pregnancy-related factors such as age, ethnic background, level of education, distance to hospital, any previous miscarriage or ectopic pregnancy and method of conception. We obtained clinical data regarding respondents’ course of pregnancy and labor from the medical records and birth registration forms that were filled out by the midwives and obstetricians. We used these data to determine the medical indications requiring a referral to obstetrician-led care, the intrapartum intervention rates, and the birth outcomes. When there was a referral from midwife-led care to obstetrician-led care during pregnancy or labor, we requested the data records of both the midwife and the obstetrician. Low-risk women whose primary choice was obstetrician-led care did not need a referral in case of a medical indication: those women were already under the supervision of an obstetrician. For this group, we reviewed all medical records to determine whether there had been medical complications or a need for care that occurred during pregnancy – based on the ‘List of Obstetric Indications’ - which would have been an indication for referral to obstetrician-led care if they were in midwife-led care. The national perinatal registry mandates that obstetricians register these medical indications in the medical records in the same way that midwives do.

### Outcome measures

Our primary outcome measure was the rate of medical indications during pregnancy. Our secondary outcome measures were the onset of labor (spontaneous, induction, planned cesarean section), intrapartum interventions (augmentation of labor, analgesia during labor, assisted vaginal birth, unplanned cesarean section, and episiotomy) and maternal and neonatal outcomes (laceration of the perineum, retention placentae, postpartum hemorrhage ≥ 1000 ml, intrapartum death- neonatal death up to 7 days - Apgar score of less than 7 at 5 minutes, resuscitation and birth weight).

### Data analysis

We analyzed the data according to the preferred place of birth indicated by women in the questionnaire before 20 weeks of gestation (midwife-led home birth, midwife-led hospital birth or obstetrician-led hospital birth), irrespective of the actual place of birth. No cases were removed from the analysis for reasons of more than 10% missing data. We compared socio-demographic and pregnancy-related characteristics among the three study groups using chi-square tests for categorical variables, analysis of variance (ANOVA) for normally distributed continuous variables and the nonparametric Kruskal-Wallis test for continuous variables that were not normally distributed. Using multiple logistic regression, we estimated odds ratios (ORs) and 95 per cent confidence intervals (95% CI) for differences in medical indications during pregnancy comparing the following groups (based on initial preferences): midwife-led home birth versus midwife-led hospital birth, midwife-led home birth versus obstetrician-led hospital birth and midwife-led hospital birth versus obstetrician-led hospital birth. In the same way we estimated ORs with 95% CI for differences in onset of labor, intrapartum interventions and maternal and neonatal outcomes. For the analysis of intrapartum interventions we excluded women with a planned cesarean section. Odds ratios were adjusted for covariates that were significantly different between the groups in the univariate analysis. We tested the regression coefficients in the model using the likelihood ratio test and the Wald statistic setting significance at α = 0.05. In more than 10% of the respondents we did not have information about the method of conception.

The study started as an RCT in 2006, but was changed into a prospective cohort study in 2007 because it was impossible to find women who would agree to be randomized for place of birth [12]. As a result, some questions were added to the questionnaire at the start of the cohort study. Because of the fact that method of conception was significantly different between the groups in the univariate analysis, we decided to include this covariate in the multivariate analysis. In addition, we carried out a sensitivity analysis without method of conception. The differences in the results were negligible (information about these results is available on request from the first author).

## Results

**Figure 1:**
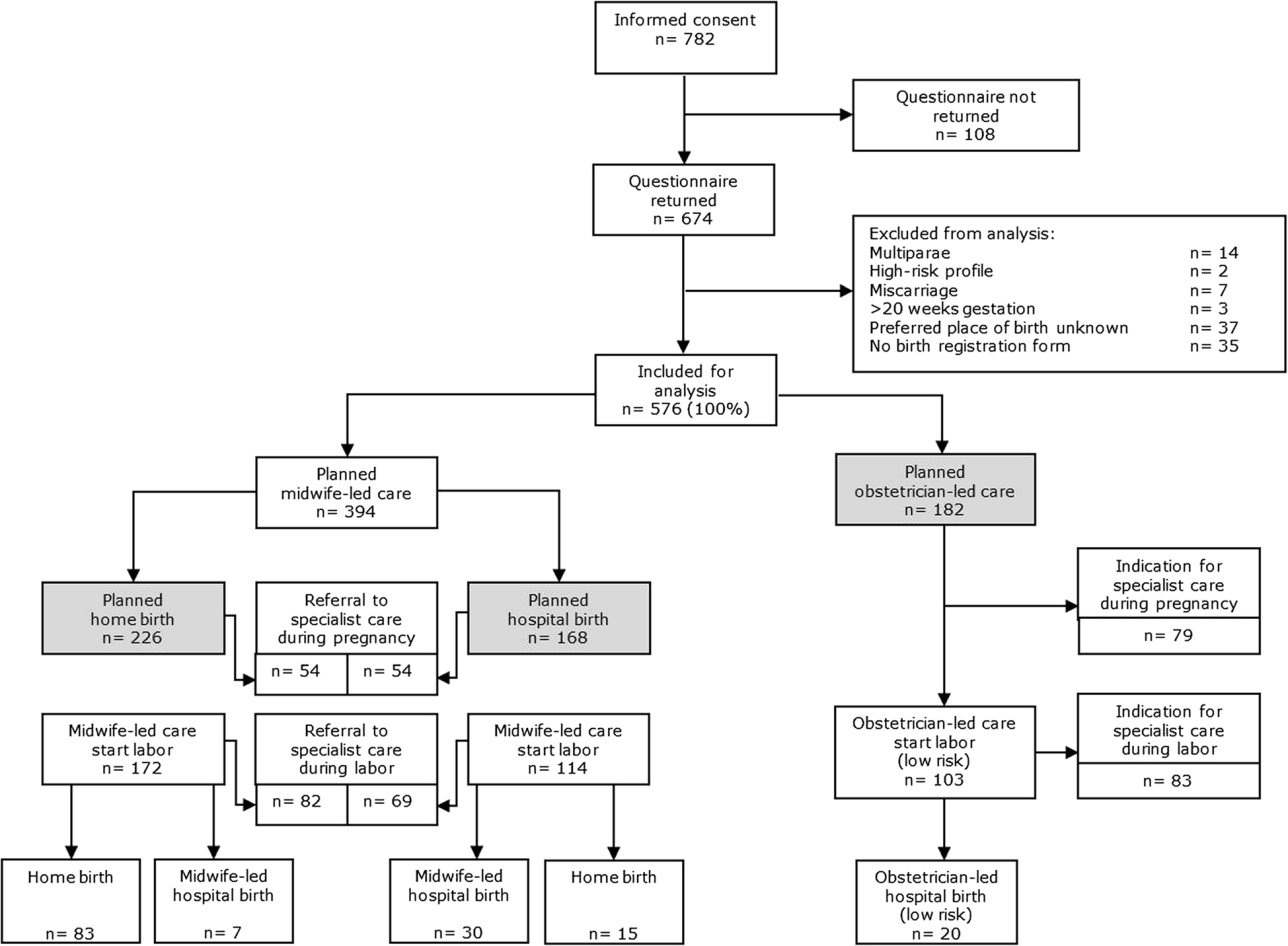
**Flowchart study population.** The highlighted parts in the flowchart were used in our analysis.

Figure 1 shows the flowchart of the study population. Of the 782 women who gave informed consent to participate in the study, 674 women completed the questionnaire, and 108 women failed to fill out the questionnaire (no reasons available), yielding a response rate of 86%. Of the 674 respondents, 26 women did not meet the inclusion criteria. Preferred place of birth was unknown in 37 cases, and birth registration forms were not complete in 35 cases. In the end, we included 576 eligible women for analysis. Of these women, 226 preferred to have a midwife-led home birth, 168 preferred a midwife-led hospital birth and 182 preferred an obstetrician-led hospital birth. Of the 576 women who started their pregnancy with a low-risk profile, 155 women (26.9%) gave birth without a diagnosed medical indication or an intervention.

### Characteristics study population

Characteristics of the three groups are presented in Table 1. No differences were observed between the two groups who preferred to have a birth with midwife-led care. However, women who preferred a birth with obstetrician-led care were slightly older (*F* (2,573) = 14.83, *p* <0.001), were more frequently pregnant after assisted reproduction (*X*^2^ (2) = 36.96, *p* <0.001), had a higher rate of previous miscarriage (*X*^2^ (2) = 28.24, *p* <0.001) and had a slightly lower median gestational age at birth (*H*(2) = 15.94, *p* < 0.001). The percentage of women with a non-Dutch background was too small in our study population to say anything about differences in ethnicity between the groups.

**Table 1:** **Characteristics of low-risk nulliparous women who initially preferred a midwife-led home or hospital birth or an obstetrician-led birth**

	**Midwife-led care**		**Obstetrician-led care**
**Variable**	**Preferred home birth n = 226 (%)**	**Preferred hospital birth n = 168 (%)**	**n = 182 (%)**
Age (years)** *mean (SD)*	28.8 (3.9)	29.1 (3.8)	30.9 (4.8)
Body Mass Index			
<18.50	10 (4.5)	5 (3.1)	7 (3.9)
18.50-24.99	139 (63.2)	115 (71.4)	121 (68.0)
≥25.00-29.99	55 (25.0)	33 (20.5)	40 (22.5)
≥30.00	16 (7.3)	8 (5.0)	10 (5.6)
Ethnic background*			
Dutch	225 (99.6)	160 (95.2)	174 (95.6)
Non-Dutch	1 (0.4)	8 (4.8)	8 (4.4)
Highest completed level of education			
Low	19 (8.4)	18 (10.7)	19 (10.4)
Middle	86 (38.1)	56 (33.3)	71 (39.0)
High	121 (53.5)	94 (56.0)	92 (50.5)
Distance to hospital (in minutes)			
0–15 minutes	170 (75.6)	135 (80.8)	135 (74.6)
>15 minutes	55 (24.4)	32 (19.2)	46 (25.4)
Method of conception**	n = 163	n = 121	n = 182
Spontaneous	154 (94.5)	110 (90.9)	132 (72.5)
Assisted Reproduction	9 (5.5)	11 (9.1)	50 (27.5)
First pregnancy**			
Yes	181 (80.1)	142 (84.5)	112 (61.9)
No^#^	45 (19.9)	26 (15.5)	69 (38.1)
Gestation at birth (days)** *mean (SD)*	278 (15)	278 (13)	274 (15)

**Significant at 1% level; *Significant at 5% level.

#previous miscarriage, ectopic pregnancy or induced abortion.

### Medical indications during pregnancy and onset of labor

Table 2 shows the overall rates of medical indications diagnosed during pregnancy, the rates per indication, and the rates of different types of onset of labor. Women who preferred a midwife-led home birth had the lowest number of medical indications (23.9%), followed by the group of women who preferred a midwife-led hospital birth (32.1%). Women who preferred an obstetrician-led hospital birth had the highest number of medical indications diagnosed during pregnancy (43.4%). The most prevalent medical indication during pregnancy in all the three groups was a hypertensive disorder. There were four cases of stillbirth: in the preferred midwife-led home birth group one unexplained stillbirth at 21 weeks of gestation, and in the preferred obstetrician-led care group, two unexplained stillbirths at 23 weeks and 39 weeks of gestation and one stillbirth due to dysmaturity at 39 weeks of gestation (birth weight: 2135 gram). Women who preferred a home or midwife-led hospital birth had lower rates of labor induction and planned cesarean section compared to women who preferred an obstetrician-led birth (10.6% and 10.7% versus 22.5% for labor induction; 4.4% and 6.5% versus 11.0% for planned cesarean). We explored the association of preferred place of birth with medical indications during pregnancy and onset of labor in a multivariate analysis adjusting for maternal age, method of conception, first pregnancy and gestational age at birth (this last variable only for onset of labor) (Table 2). The likelihood of a diagnosis of a medical indication during pregnancy was significantly reduced in the group of women who preferred a home birth compared to women who preferred an obstetrician-led birth (adjusted OR 0.41, 95% CI 0.25–0.66). The same trend was observed for women who preferred a home birth compared to women who preferred a midwife-led hospital birth and for women who preferred a midwife-led hospital birth compared to women who preferred an obstetrician-led birth, but these results were not statistically significant (adjusted OR 0.64, 95% CI 0.38–1.09 resp. 0.64, 95% CI 0.39–1.04). Women who preferred a birth with midwife-led care, either at home or in hospital, were significantly less likely to have their labor induced compared to women who preferred a birth with obstetrician-led care (adjusted OR 0.51, 95% CI 0.28-0.95 for the home birth group and adjusted OR 0.42, 95% CI 0.21-0.85 for the midwife-led hospital group). The odds of women having a planned cesarean section were not significantly different among groups.

**Table 2:** **Association between the initial preferred birth setting and medical indications during pregnancy and onset of labor**

	**Midwife-led care**		**Obstetrician-led care**	**OR (adjusted)** ^**a**^	**OR (adjusted)** ^**a**^	**OR (adjusted)** ^**a**^
				**(95% CI)**	**(95% CI)**	**(95% CI)**
	**Preferred home birth**	**Preferred hospital birth**	**n = 182 (%)**	**Preferred MFL home birth vs. Preferred MFL hospital birth**	**Preferred MFL home birth vs. Preferred OBL hospital birth**	**Preferred MFL hospital birth vs. Preferred OBL hospital birth**
	**n = 226 (%)**	**n = 168 (%)**				
Medical indications (overall)^a^	54 (23.9)	54 (32.1)	79 (43.4)	0.64 (0.38 – 1.09)	0.41 (0.25 – 0.66)	0.64 (0.39 – 1.04)
Hypertensive disorders	19 (8.4)	15 (8.9)	27 (14.8)			
(Suspected) IUGR	1 (0.4)	5 (3.0)	8 (4.4)			
Diabetes	-	1 (0.6)	3 (1.6)			
Congenital anomalies	1 (0.4)	-	1 (0.5)			
Stillbirth	1 (0.4)	-	3 (1.6)			
Malposition incl. breech	7 (3.1)	10 (6.0)	13 (7.1)			
Placental problems , blood loss	1 (0.4)	1 (0.6)	4 (2.2)			
(Threatening) preterm birth	8 (3.5)	9 (5.4)	6 (3.3)			
Post-term pregnancy	11 (4.9)	9 (5.4)	6 (3.3)			
Other	5 (2.2)	4 (2.4)	8 (4.4)			
Onset of labor^a,^ ^b^						
Spontaneous	192 (85.0)	139 (82.7)	121 (66.5)			
Induction	24 (10.6)	18 (10.7)	41 (22.5)	1.23 (0.57 – 2.64)	0.51 (0.28 – 0.95)	0.42 (0.21 – 0.85)
Planned cesarean	10 (4.4)	11 (6.5)	20 (11.0)	0.74 (0.25 – 2.21)	0.41 (0.16 – 1.04)	0.55 (0.22 – 1.41)

^a^Adjusted for maternal age, method of conception, first pregnancy (previous miscarriage or ectopic pregnancy), ^b^gestational age at birth. OR = Odds Ratio; CI 95% = confidence interval 95%.

MFL = Midwife-led; OBL = Obstetrician-led.

### Intrapartum interventions and maternal outcomes

The frequency of intrapartum interventions and maternal outcomes are listed in Table 3. Overall, women who preferred to have a home birth experienced the lowest intervention rates, except for episiotomy: 56.3% versus 51.9% for women who preferred a midwife-led hospital birth. Women who preferred a birth with obstetrician-led care had the highest intervention rates with the exception of unplanned cesarean sections (13.6% versus 15.3% for women who preferred a midwife-led hospital birth). The number of perineal tears was lowest in the group preferring obstetrician-led care (17.3% versus 23.9% and 31.1% for women who preferred a home birth and women who preferred a midwife-led hospital birth respectively). On the other hand, the number of episiotomies was the highest in the preferred obstetrician group with a percentage of 68.3%. In a multivariate analysis adjusting for maternal age, method of conception, first pregnancy, gestational age at birth and medical indications during pregnancy, women with a preference for a home birth were less likely to have augmentation of labor (adjusted OR 0.54 95% CI 0.32-0.93), narcotic analgesia (adjusted OR 0.41 95% CI 0.21-0.79), and epidural analgesia (adjusted OR 0.32 95% CI 0.18-0.56) compared to women with a preference for an obstetrician-led birth (Table 3). Women who preferred a midwife-led hospital birth were less likely to experience epidural analgesia (adjusted OR 0.34 95% CI 0.19-0.62) and an episiotomy (adjusted OR 0.49 95% CI 0.30-0.81) compared to women who preferred an obstetrician-led birth. We observed no significant differences in any of the intrapartum interventions between the home birth group and the midwife-led hospital group. In addition, the odds of assisted vaginal birth, unplanned cesarean section and the maternal outcomes were not statistically different between all the three groups.

**Table 3:** **Association between initial preferred birth setting and intrapartum interventions and maternal outcomes**

	**Midwife-led care**		**Obstetrician-led care**	**OR (adjusted)** ^**a**^	**OR (adjusted)** ^**a**^	**OR (adjusted)** ^**a**^
				**(95% CI)**	**(95% CI)**	**(95% CI)**
**Interventions/maternal outcomes**	**Preferred home birth**	**Preferred hospital birth**	**n = 182 (%)**	**Preferred MFL home birth vs. Preferred MFL hospital birth**	**Preferred MFL home birth vs. Preferred OBL hospital birth**	**Preferred MFL hospital birth vs. Preferred OBL hospital birth**
	**n = 226 (%)**	**n = 168 (%)**				
Augmentation of labor	n = 192	n = 137	n = 120			
No	133 (69.3)	83 (60.6)	61 (50.8)			
Oxytocin or prostaglandins	59 (30.7)	54 (39.4)	59 (49.2)	0.79 (0.46 – 1.35)	0.54 (0.32 – 0.93)	0.69 (0.40 – 1.21)
Analgesia during labor	n = 215	n = 155	n = 160			
No	156 (72.6)	96 (61.9)	66 (41.3)			
Narcotic analgesia	25 (11.6)	33 (21.3)	35 (21.9)	0.56 (0.28 – 1.09)	0.41 (0.21 – 0.79)	0.74 (0.39 – 1.38)
Epidural analgesia	34 (15.8)	26 (16.8)	59 (36.9)	0.94 (0.48 – 1.83)	0.32 (0.18 – 0.56)	0.34 (0.19 – 0.62)
Mode of birth	n = 216	n = 157	n = 162			
Spontaneous vaginal	161 (74.5)	104 (66.2)	103 (63.6)			
Assisted vaginal (VE/FE)	37 (17.1)	29 (18.5)	37 (22.8)	0.94 (0.49 – 1.83)	0.83 (0.45 – 1.53)	0.88 (0.46 – 1.68)
Cesarean (unplanned)	18 (8.3)	24 (15.3)	22 (13.6)	0.48 (0.23 – 1.02)	0.75 (0.35 – 1.59)	1.55 (0.76 – 3.17)
Episiotomy	111 (56.3)	70 (51.9)	95 (68.3)	1.40 (0.86 – 2.29)	0.69 (0.44 – 1.10)	0.49 (0.30 – 0.81)
Perineum	n = 197	n = 135	n = 139			
Intact	39 (19.8)	23 (17.0)	20 (14.4)			
Tear	47 (23.9)	42 (31.1)	24 (17.3)	0.72 (0.41 – 1.26)	1.58 (0.87 – 2.87)	2.21 (1.18 – 4.12)
First- or second degree tear	46	34	22			
Third- or fourth degree tear	1	8	2			
Retained placenta	4 (2.0)	3 (2.2)	8 (5.7)	NT	NT	NT
Postpartum hemorrhage	n = 221	n = 160	n = 176			
<500 cc	158 (71.5)	108 (67.5)	109 (61.9)			
≥500 - < 1000 cc	50 (22.6)	42 (26.3)	48 (27.3)			
≥1000 cc	13 (5.9)	10 (6.3)	19 (10.8)	0.82 (0.32 – 2.08)	0.57 (0.25 – 1.30)	0.70 (0.30 – 1.63)

^a^Adjusted for maternal age, method of conception, first pregnancy (previous miscarriage or ectopic pregnancy), gestational age at birth, medical indications pregnancy. OR = Odds Ratio; CI 95% = confidence interval 95%.

MFL = Midwife-led; OBL = Obstetrician-led.

### Neonatal outcomes

Neonatal death, Apgar score of less than 7 after 5 minutes and resuscitation were rare in all three groups. Therefore, it was not meaningful to perform a statistical test. We found no significant differences in birth weight between the three groups (Table 4).

**Table 4:** **Association between initial preferred birth setting and neonatal outcomes**

	**Midwife-led care**		**Obstetrician-led care**	**OR (adjusted)** ^**a**^	**OR (adjusted)** ^**a**^	**OR (adjusted)** ^**a**^
				**(95% CI)**	**(95% CI)**	**(95% CI)**
	**Preferred home birth**	**Preferred hospital birth**	**n = 182 (%)**	**Preferred MFL home birth vs. Preferred MFL hospital birth**	**Preferred MFL home birth vs. Preferred OBL hospital birth**	**Preferred MFL hospital birth vs. Preferred OBL hospital birth**
	**n = 226 (%)**	**n = 168 (%)**				
Neonatal death (dp – 7 dgn)	-	-	-			
Apgar score < 7 at 5 min	4 (1.8)	3 (1.8)	4 (2.2)	NT	NT	NT
Resuscitation	1 (0.4)	-	-			
Birth weight *mean (SD)*	3365 (543)	3401 (575)	3210 (555)			
2500 – 3999 gram	195 (86.3)	135 (80.4)	155 (85.2)			
<2500 gram	11 (4.9)	10 (6.0)	15 (8.2)	0.68 (0.16 – 2.84)	0.83 (0.22 – 3.12)	1.23 (0.36 – 4.20)
≥4000 gram	20 (8.8)	23 (13.7)	12 (6.6)	0.69 (0.32 – 1.52)	1.02 (0.44 – 2.40)	1.47 (0.63 – 3.46)

^a^Adjusted for maternal age, method of conception, first pregnancy (previous miscarriage or ectopic pregnancy), gestational age at birth, medical indications pregnancy. OR = Odds Ratio; CI 95% = confidence interval 95%.

MFL = Midwife-led; OBL = Obstetrician-led.

## Discussion

The aim of our study was to explore whether the initial preferred place of birth at the onset of pregnancy and model of care are associated with differences in the course of pregnancy and intrapartum interventions and birth outcomes. We found that low-risk nulliparous women who preferred a home birth were less likely to experience a medical indication during pregnancy compared to women who preferred a birth with obstetrician-led care. Furthermore, preferring a birth with midwife-led care – both at home and in hospital - was associated with lower rates of induced labor and lower rates of epidural analgesia. In our study, preferred place of birth was not associated with differences in mode of birth.

The difference we found in medical indications during pregnancy in relation to preferred place of birth and model of care is intriguing, because one would not expect the preferred place or model of care to influence the likelihood of developing, for instance, a hypertensive disorder. In principle, the same care is given during pregnancy, but it is likely that each professional acts from his or her own paradigm. The difference in medical indications between midwife-led and obstetrician-led care could be a matter of difference in clinical judgment between the maternity care providers. It is recognized in medical sociology that differences in opinion or judgment between care providers are part of a wider phenomenon, namely that concepts of health and illness are socially constructed and differ between care providers [14,15]. Another, somewhat weaker, explanation mentioned by Eskes [16] is that some intuitive form of self-selection regarding medical complications occurs among low-risk pregnant women. However, this has never been properly investigated. Considering induction of labor and intrapartum interventions, our results are in line with previous studies showing that midwife-led care for low-risk women reduces the risk of some interventions when compared to obstetrician- or physician-led care [1,4,8,9]. Reime et al. [17] reported that obstetricians were more attached to technology and interventions, including inductions, compared to midwives. However, our results are based on the initial preferred place of birth at the beginning of pregnancy (intention-to-treat), instead of the planned place at the onset of labor. This introduces the possibility that differences in findings between the groups were not only attributable to model of care or care provider, but also to attitudes and characteristics of the women. Van der Hulst et al. [7] observed that the more receptive women’s attitude was toward medical technology, the more likely women were to opt for a hospital birth, and the more likely it was they would experience an obstetrical intervention. In a previous study where we explored women’s preferences for aspects of intrapartum care regarding planned place of birth we reported that women with a preference for a hospital birth – both midwife-led and obstetrician-led – found the possibility of pain relief treatment much more important compared to women with a preference for a home birth [18]. This could be an explanation for the fact that women who preferred a birth at home –irrespective of their actual place of birth- experienced lower rates of narcotic and epidural analgesia. The rates of assisted vaginal births and cesarean sections in this study are comparable to the national data of nulliparous women from 2012 (16.4% assisted vaginal birth and 17.7% caesarean section) [19]. Our study shows no differences in association between preferred place of birth and mode of birth. The fact that the Netherlands has low rates of assisted births and cesarean sections in general probably plays a role in this. Overall rates in 2012 for assisted vaginal births and cesarean sections (planned and unplanned combined) were 9.2% and 16.3%, respectively [19].

### Strengths and limitations

Most of the studies about place of birth have been done in countries where giving birth outside the hospital is not always available or more difficult to arrange. It can be assumed, then, that women in those countries who planned a home birth belong to a select and highly motivated group. This difference in populations may influence the results of studies in those countries. In the Netherlands, however, both home and hospital are seen as a safe and normal place to give birth. A main advantage of our study is its prospective design, which enables us to explore the association between preferred place of birth and the course of both pregnancy and childbirth. Another advantage is that we were able to include low-risk women with a preference for three different settings.

Our study has some limitations. The inclusion period for women with a preference for obstetrician-led care was much longer than we expected. It seems there were fewer low-risk women with a preference for obstetrician-led care than we initially assumed, indicating that obstetrician-led care for low-risk women is uncommon in the Netherlands. Another limitation of our study is the possibility of selection bias. We had little direct control over the inclusion processes in midwifery practices and hospitals, and thus we do not know the exact number of women who were eligible during the period of recruitment. Furthermore, we do not have information about characteristics of the women who were eligible for the study but refused to participate. The reason most often given by women for not participating in the study after the researchers called them was a lack of time. In our study, the number of women with a low level of education was probably smaller compared to the Dutch population [20]. It is possible that women with a lower level of education more often refused to participate. Level of education may have influenced the likelihood of diagnosing a medical indication during pregnancy or an intrapartum intervention. However, there was no significant difference in level of education between the three study groups. The percentage of women with a non-Dutch background was also small in our study population: 3.1% in total, as compared with 25.3% of all nulliparous women in the Netherlands in 2012 [19]. This is a result of the fact that only women who understood the Dutch language could be enrolled in the study. For these reasons, it is unclear to what extent our results apply to lower-educated women and ethnic minority populations in the Netherlands.

## Conclusions

Our study demonstrates significant differences in the course of pregnancy and labor in relation to *preferred* place of birth, as showed by the fewest number of diagnosed medical indications during pregnancy and the fewest intrapartum interventions among women who preferred a home birth. Although some differences can be attributed to the eventual model of care – *i.e. midwife-led or obstetrician-led -* we suggest that characteristics and attitudes of women also play an important role. Maternity care providers should take this into account. For a better understanding regarding the choice for place of birth and the consequences of that for pregnancy and childbirth, future research should focus more on these characteristics and attitudes. In addition, we should explore the process of decision making around determining indications for specialist care or interventions, both from the perspective of the care providers and the women.
